# Association of peripheral anterior synechiae with anterior segment parameters in eyes with primary angle closure glaucoma

**DOI:** 10.1038/s41598-021-93293-7

**Published:** 2021-07-06

**Authors:** Yunhua Loo, Tin A. Tun, Eranga N. Vithana, Shamira A. Perera, Rahat Husain, Tina T. Wong, Tin Aung, Monisha E. Nongpiur

**Affiliations:** 1 Singapore National Eye Centre, Singapore, Singapore; 2 Singapore Eye Research Institute, The Academia, 20 College Road, Discovery Tower Level 6, Singapore, 169856 Singapore; 3 Duke-NUS Medical School, Singapore, Singapore; 4 Department of Ophthalmology, Yong Loo Lin School of Medicine, National University of Singapore, Singapore, Singapore

**Keywords:** Eye diseases, Optic nerve diseases

## Abstract

To investigate the association of peripheral anterior synechiae (PAS) with intraocular pressure (IOP) and anterior-segment parameters in subjects with primary angle-closure glaucoma (PACG). A total of 267 subjects with PACG were recruited and underwent gonioscopy and anterior-segment optical coherence tomography (ASOCT). Customized software was used to measure ASOCT parameters, including angle opening distance (AOD750) and trabecular-iris-space-area (TISA750) at 750 µm from the scleral spur, anterior chamber depth, width, area and volume (ACD, ACW, ACA, ACV), iris thickness (IT750), iris area (IAREA), and lens vault (LV). Presenting IOP was defined as the first IOP reading before the initiation of IOP-lowering treatment. The mean age of the 267 subjects was 67.0 ± 8.9 years, 140 (52.4%) were male, and 246 (92.1%) were of Chinese ethnicity. PAS was present in 122 (45.7%) subjects, and was most frequently found in the superior quadrant (79.5%). Subjects with PAS had greater presenting IOP (28.7 ± 12.9 vs 22.4 ± 9.7 mmHg, p < 0.001), narrower AOD750 (p < 0.001), smaller TISA750 (p < 0.001), ACD (p = 0.04), ACA (p = 0.02), ACV (p = 0.01) and larger LV (p = 0.01) compared to PACG eyes without PAS. No significant differences were noted for iris parameters. A multivariate logistic regression analysis showed that higher presenting IOP (β = 0.20, p < 0.001), worse visual field mean deviation (β = − 0.20, p = 0.01) and narrower AOD750 (β = − 0.25, p = 0.03) were the only parameters that significantly correlated with the extent of PAS in clock hours. Almost one-half of the subjects with PACG demonstrated PAS; these eyes were associated with higher presenting IOP, smaller anterior segment dimensions and more severe disease.

## Introduction

Primary angle closure glaucoma (PACG) is a major cause of blindness worldwide, with a significantly higher prevalence in Asian populations as compared to Caucasian and Black population^1,2^. Compared to primary open angle glaucoma (POAG) subjects, patients with PACG have a higher rate of severe visual loss^3,4^. In PACG, closure of the anterior chamber leads to reduction in aqueous outflow, causing raised intraocular pressure (IOP) and subsequent optic nerve damage and visual field loss.

Peripheral anterior synechiae (PAS) are adhesions of the peripheral iris to the angular structures of the anterior chamber and can be observed in a variety of conditions including uveitis, neovascular glaucoma, iridocorneal endothelial syndrome and following ocular trauma or surgeries. In primary angle closure disease, it is speculated that intermittent episodes of transient iridotrabecular contact can become more permanent and lead to the formation of PAS. Several studies have reported the association of PAS with ocular parameters including IOP and angle width. In a large population-based study of 2,045 subjects, Chong et al. showed that the presence of PAS increased with the number of quadrants of closed angles as seen on gonioscopy as well as on anterior segment optical coherence tomography (ASOCT)^5^. Su et al. have also shown in ASOCT studies that eyes with PAS had smaller angle dimensions such as angle open distance (AOD), angle recess area (ARA) and trabecular iris space area (TISA)^6^ compared to those without PAS. Improvements in ASOCT technology have led to the identification of several imaging-based anterior segment parameters that are associated with an increased risk for angle closure, such as smaller anterior chamber width^7^, area and volume (ACW, ACA and ACV)^8^, thicker iris with greater iris cross-sectional area^9^ and increased lens vault (LV)^10^. The association between the recently described angle closure risk factors and PAS in eyes with PACG is, however, currently not known. The aim of this study was to examine the extent and distribution of PAS in eyes with PACG and the association of PAS with quantitative parameters of the anterior segment including the anterior chamber, lens and iris. A better understanding of the determinants of PAS formation in PACG eyes may be useful clinically in the management of the disease.

## Materials and methods

Subjects diagnosed with PACG were prospectively recruited from the glaucoma clinics of the Singapore National Eye Centre. All subjects had undergone laser peripheral iridotomy (LPI) prior to recruitment into the study, but we excluded those who had undergone any other surgical procedure including argon laser peripheral iridoplasty (ALPI). Patients with a history of previous attack of acute angle closure, secondary angle closure, trauma, and ocular inflammation were not included. Each subject underwent a standardized ophthalmic examination that included an assessment of the visual acuity, slit lamp examination, stereoscopic evaluation of the optic disc, IOP measurement with Goldmann applanation tonometry, and visual field testing. Gonioscopy was performed using a Goldmann 2-mirror lens by an experienced examiner (MEN) under dark conditions at high magnification (× 16). The presence of PAS was determined by indentation gonioscopy using a Sussman 4 mirror lens (Ocular Instruments Inc., Bellevue, WA) and was defined as abnormal adhesions of the iris to the angle that were at least half a clock hour in width and were present to the level of the anterior trabecular meshwork or higher. The extent of PAS across the superior, inferior, nasal and temporal quadrants was documented in clock hours. PACG was defined as eyes with narrow angles (at least 180° of the posterior pigmented trabecular meshwork was not visible on non-indentation gonioscopy in the primary position of gaze) with evidence of glaucomatous optic neuropathy (GON, defined as vertical cup-to-disc ratio [CDR] more than or equal to 0.7, CDR asymmetry of more than 0.2, and/or the presence of focal notching with compatible visual field loss on static automated perimetry [SITA Standard algorithm with a 24-2 test pattern; Humphrey Visual Field Analyzer II; Carl Zeiss Meditec, Dublin, CA]). Axial length (AL) and lens thickness (LT) measurements were also obtained using an A-scan ultrasonography (Model US-800; Nidek Co., Ltd., Tokyo, Japan).

Presenting IOP was defined as the first IOP reading measured by Goldmann applanation tonometry before the initiation of IOP-lowering treatment (medical or laser). These data were extracted from the case records. Severity of glaucoma was determined from the visual field mean deviation (MD), and was categorized as mild (MD > − 6 dB), moderate (MD − 6.01 to − 12 dB), and severe (< − 12.01 dB)^11^.

### Anterior segment optical coherence tomography (ASOCT) imaging and analysis

ASOCT (Visante; Carl Zeiss Meditec Dublin, CA) imaging was performed for all subjects by a single operator (TAT), under standardized dark conditions (0 lx). The scans were centred on the pupil and taken along the horizontal axis (nasal–temporal angles at 0°–180°) using the standard anterior segment single-scan protocol. In order to obtain the best quality scans, the examiner adjusted the saturation and noise, and optimized the polarization for each scan during the examination. The examiner chose the best image, with no motion or image artefacts caused by the eyelids. These images were processed using customized software, the Zhongshan Angle Assessment Program (ZAAP; Guangzhou, China) by a single experienced observer (MEN) who was masked to clinical data. The only observer input was to determine the location of the scleral spurs, following which the algorithm automatically calculated the anterior segment parameters. Images with poor quality, indeterminate scleral spur and software delineation errors were excluded. The following parameters were measured: angle opening distance at 750 µm from the scleral spur (AOD750); trabecular–iris space area at 750 µm from the scleral spur (TISA750); angle recess area (ARA), iris thickness at 750 µm from the scleral spur (IT750); iris curvature (ICURV), iris area (IAREA), anterior chamber depth (ACD), width (ACW), area (ACA), volume (ACV), and lens vault (LV)^12^. For the anterior chamber angle and iris parameters, the mean of the nasal and temporal values was computed.

### Statistical analysis

Statistical analysis was performed using a commercially available statistical software package (SPSS for Windows, version 22.0; IBM-SPSS, Chicago, IL, USA). One eye of each subject was analysed. If both eyes of a single subject were found to have same diagnosis, the more severe eye based on visual field MD was chosen. If both eyes had similar diagnosis and severity, then the study eye was randomly chosen by tossing a coin. Comparison of the proportion of PAS between the groups was assessed by chi-square test. Differences in mean values of parameters between two groups were examined using the independent samples Student t-test. Correlation between continuous data variables was analysed using the Pearson correlation test (2-sided). Multivariate linear regression analysis was performed to identify parameters that correlated significantly with the extent of PAS formation. Significance was set at p < 0.05 for this study. A priori power calculations were performed, using G*Power software version 3.1.9.6, to evaluate the sample size required to detect statistically significant differences in the primary outcome measures, namely IOP and ASOCT parameters. The sample size was calculated based on the difference between two independent means for eyes with and without PAS using a one tail test, with significance level alpha set at 0.05 and power set at 0.80.

### Ethics approval

Approval for the study was granted by the Institutional Review Board of the SingHealth Centralized Institutional Review Board. The study was conducted in accordance with the tenets of the Declaration of Helsinki.

### Consent to participate

Informed consent was obtained from all individual participants included in the study,

### Consent for publication

All participants have consented for their data to be published.

## Results

A total of 267 subjects with PACG were included in this study. The demographic and ocular characteristics are shown in Table 1. The majority of patients were of Chinese ethnicity (246, 92.1%) and male (140, 52.4%). The average age of all subjects was 67.0 ± 8.9 years and presenting IOP was 25.2 ± 11.6 mmHg.

**Table 1:** Demographic and ocular characteristics of the subjects.

Total no. of patients, N	267
Age, years	67.0 ± 8.9
Male, N (%)	140 (52.4)
Race (Chinese), N (%)	246 (92.1)
Presenting IOP, mmHg	25.2 ± 11.6
Vertical cup-to-disc ratio	0.79 ± 0.11
Mean deviation, dB	− 12.20 ± 8.61
Lens thickness, mm	4.49 ± 0.84
Axial length, mm	23.03 ± 0.93

*IOP* intraocular pressure.

Table 2 shows the distribution and extent of PAS by quadrants across the PACG severity spectrum. Peripheral anterior synechiae was present in 122 (45.7%) subjects and was most frequently found in the superior quadrant (79.5%), followed by the inferior (45.1%), nasal (43.4%) and temporal (37.7%) quadrants respectively. In terms of the absolute magnitude, PACG eyes that had PAS demonstrated on average 3.84 ± 2.64 clock hours of PAS, with the superior quadrant having the highest amount of PAS (1.64 ± 1.11 clock hours), followed by the nasal, inferior and temporal quadrants respectively. Subjects with severe PACG were more likely to have PAS (55.2%) compared to subjects with mild or moderate PACG (p = 0.02 for both). They also demonstrated significantly more clock hours of PAS in the superior quadrant compared to counterparts with mild or moderate PACG (p = 0.046).

**Table 2:** Distribution and extent of PAS across PACG disease severity (N = 267).

	All eyes	Mild PACG N = 80	Moderate PACG N = 71	Severe PACG N = 116	p-value
**Proportion of subjects with PAS, N (%)**					
	122 (45.7)	31 (38.8)	27 (38.0)	64 (55.2)	0.02*
Superior PAS, N (%)	97 (79.5)	22 (71.0)	21 (77.8)	54 (84.4)	0.31
Nasal PAS, N (%)	53 (43.4)	13 (41.9)	9 (33.3)	31 (48.4)	0.41
Inferior, N (%)	55 (45.1)	17 (54.8)	9 (33.3)	29 (45.3)	0.26
Temporal, N (%)	46 (37.7)	10 (32.3)	11 (40.7)	25 (39.1)	0.76
**Total Clock hours of PAS**					
	3.84 ± 2.64	3.45 ± 2.16	3.15 ± 2.32	4.31 ± 2.91	0.10
Superior	1.64 ± 1.11	1.32 ± 1.11	1.44 ± 1.09	1.87 ± 1.08	0.046**
Nasal	0.85 ± 1.12	0.77 ± 1.02	0.57 ± 0.95	1.00 ± 1.21	0.23
Inferior	0.75 ± 1.00	0.87 ± 0.99	0.59 ± 0.97	0.77 ± 1.04	0.58
Temporal	0.60 ± 0.89	0.48 ± 0.77	0.54 ± 0.72	0.68 ± 1.00	0.56

*PAS* peripheral anterior synechiae, *PACG* primary angle closure glaucoma.

*Mild vs Severe PACG and Moderate vs Severe PACG = 0.02.

**Mild vs Severe PACG = 0.07.

Comparison of the presenting IOP and ASOCT parameters between subjects with and without PAS is shown in Table 3. Subjects with PAS had greater presenting IOP (28.7 ± 12.9 vs 22.4 ± 9.7 mmHg in eyes without PAS, p < 0.001). ASOCT images were available for a total of 240 subjects, of which 26 were excluded due to poor quality images which had low resolution that precluded distinct visualization of the anterior chamber angle, iris boundaries and lens (N = 10), indeterminate scleral spur (N = 4), and software delineation errors (N = 12). Eyes with PAS demonstrated significantly narrower AOD (p < 0.001), smaller TISA (p < 0.001), smaller ACD (p = 0.04), ACA (p = 0.02), ACV (p = 0.01) and larger LV (p = 0.01) compared to those without PAS. However, no significant differences were noted for iris parameters. A priori power analysis was performed to evaluate the sample size required to detect statistical significance for the following parameters (with the effect size in parentheses): IOP (0.55), AOD750 (0.46), TISA750 (0.72), ACV (0.339), ACA (0.340), LV (0.360) and ACD (0.32). The required sample size was calculated to be 42, 61, 25, 109, 108, 97 and 120 respectively. Since our study had 122 eyes with PAS and 145 eyes without PAS, this indicates that the study is sufficiently powered.

**Table 3:** Comparison of the presenting IOP and ASOCT parameters in eyes with and without peripheral anterior synechiae in eyes with primary angle closure glaucoma.

	PAS present N = 122	PAS absent N = 145	p-value
Presenting IOP, mmHg	28.7 ± 12.9	22.4 ± 9.7	< 0.001
Mean deviation, dB	− 13.5 ± 9.1	− 11.1 ± 8.1	0.03
Analysable ASOCT, N = 214	N = 93	N = 121	
AOD750, mm	0.21 ± 0.11	0.26 ± 0.11	< 0.001
TISA750, mm	0.10 ± 0.05	0.14 ± 0.06	< 0.001
ACD, mm	2.10 ± 0.32	2.19 ± 0.30	0.04
IT750, mm	0.46 ± 0.09	0.45 ± 0.08	0.46
IAREA, mm^2^	1.62 ± 0.31	1.65 ± 0.24	0.41
ACW, mm	11.4 ± 0.4	11.4 ± 0.4	0.35
ACA, mm^2^	15.3 ± 2.6	16.2 ± 2.7	0.02
ACV, mm^3^	98.7 ± 20.2	105.7 ± 21.1	0.01
LV, µm	923 ± 276	822 ± 286	0.01

*IOP* intraocular pressure, *ASOCT* anterior segment optical coherence tomography, *ACD* anterior chamber depth, *AOD750* angle opening distance at 750 µm from the scleral spur, *TISA750* trabecular–iris space area at 750 µm from the scleral spur, *IT750* iris thickness at 750 µm from the scleral spur, *IAREA* iris area, *ACW* anterior chamber width, *ACA* anterior chamber area, *ACV* anterior chamber volume, *LV* lens vault.

The correlations between the extent of PAS and ocular parameters are shown in the online supplementary Table S1. Significant correlations were observed between the extent of PAS and the presenting IOP and AOD750. Eyes with greater amplitude of PAS presented with significantly higher IOP (correlation coefficient [r] = 0.43, p < 0.001) and also exhibited smaller AOD750 (r = − 0.29, p < 0.001). Modest correlations were also found between the extent of PAS and visual field MD, ACD, ACA and LV, whereby eyes with greater amounts of PAS demonstrated worse visual field MD (r = − 0.23, p < 0.001), shallower ACD (r = − 0.16, p = 0.02), smaller ACA (r = -0.15, p = 0.03) and larger LV (r = 0.18, p = 0.007).

Table 4 presents univariate and multivariate linear regression analysis for the variables associated with the extent of PAS, after adjusting for age and gender. Higher presenting IOP (β = 0.20, p < 0.001), mean deviation (β = − 0.20, p = 0.01) and narrower AOD750 (β = − 0.25, p = 0.03) were the only parameters that correlated significantly with the extent of PAS.

**Table 4:** Factors associated with peripheral anterior synechiae in eyes with primary angle closure glaucoma.

Predictor Variable	Univariate linear regression*		Multiple linear regression	
	β	P value	β	P value
Age	− 0.07	0.009	− 0.10	0.21
Gender (Male)	0.02	0.88	− 0.05	0.54
Presenting IOP	0.41	< 0.001	0.20	0.03
Mean deviation	− 0.22	0.001	− 0.20	0.01
ACD	− 0.19	0.006		
ACA	− 0.18	0.009	− 0.03	0.76
ACW	− 0.03	0.63		
AOD750	− 0.30	< 0.001	− 0.25	0.008
IT750	0.06	0.38		
IArea	− 0.13	0.06	− 0.09	0.20
Icurv	− 0.10	0.14		
Lens Vault	0.24	0.001	0.08	0.42

* adjusted for age and gender.

*IOP* intraocular pressure, *ACD* anterior chamber depth, *AOD750* angle opening distance at 750 µm from the scleral spur, *TISA750* trabecular–iris space area at 750 µm from the scleral spur, *IT750* iris thickness at 750 µm from the scleral spur, *IAREA* iris area, *Icurv* iris curvature, *ACW* anterior chamber width, *ACA* anterior chamber area, *ACV* anterior chamber volume, *LV* lens vault.

## Discussion

In this cross-sectional study, we evaluated the distribution of PAS in subjects with PACG. Our findings showed that approximately half of PACG eyes have PAS, and with a greater proportion in eyes with more severe disease. Eyes with PAS were characterized by smaller anterior chamber dimensions, narrower angles and large LV, however, multivariate analysis showed that the extent of PAS was significantly associated with only narrower anterior chamber angles, higher presenting IOP and worse visual field MD (dB).

Consistent with several previous studies, we also found that PAS was most commonly observed in the superior quadrant^13–16^. The predilection for PAS in the superior quadrant has been postulated to be due to several reasons. Phillips postulated that the relatively narrower angle anatomy of superior quadrant compared to other parts of the eye predisposes PAS formation^13–16^. The sectoral variations in angle depth in turn could be a result of sectoral differences in the position of the iris origin from the ciliary body, sectoral discrepancies in angle development during embryonic differentiation, differences in lens orientation, pupil decentration and irregular peripheral flattening of the cornea. Progressive shallowing of the anterior chamber with preferential narrowing in the superior quadrant has been observed in prospective studies in Japanese patients^17^. In addition, it is important to consider the potential effect of LPI on the development and/or progression of PAS. In our practice, LPI is typically sited in the superior quadrant. Unfortunately, however, the exact site of the LPI was not documented in our study. We hypothesize that localised inflammation post-laser iridotomy may also contribute to PAS formation but longitudinal studies would be needed to evaluate whether the position of the LPI influences localised PAS formation or its expansion.

Several studies that have utilised ASOCT imaging to evaluate anterior segment morphology have shown that PACG eyes are more likely to have smaller anterior chamber dimensions, large LV and thick iris^7–10,18,19^. While it is widely accepted that PAS can lead to obstruction of aqueous outflow and contribute to glaucomatous nerve damage in PACG, the structural relationship between PAS and ASOCT parameters, particularly the recently identified risk factors for angle closure remained largely unexplored. Most studies have only evaluated parameters related to the anterior chamber angle dimensions. In their evaluation of 203 subjects including 76 with primary angle closure (PAC) or PACG, Su et al.^6^ found that eyes with PAS demonstrated narrower angle width (smaller AOD, ARA and TISA). Similarly, using ultrasound biomicroscopy (UBM), Yoo and colleagues showed that the presence of PAS correlated with shorter trabecular-ciliary process distance in the superior quadrant but this study was performed in only PAC eyes^20^. The findings from our study performed in a homogenous population of PACG eyes, concurs with previous studies. However, in addition to narrower angle width, we also observed that eyes with PAS had smaller anterior chamber depth, area and a larger LV and these parameters also correlated significantly with the extent of PAS in clock hours. Our data are in line with previous work from Gazzard et al. which showed increased risk of PAS formation in Asian eyes that have shallower anterior chambers as a result of thicker and more anteriorly-placed lenses (ARVO abstract)^21^. Whilst a shallow anterior chamber is the cardinal risk factor for angle closure, several studies have also illustrated that characteristics of the iris, such as peripheral iris thickness also contribute to iridotrabecular contact in angle closure eyes^9,22,23^. However, in our data, quantitative iris parameters as determined from ASOCT including iris thickness and area, did not correlate with the presence of PAS in PACG eyes.

The development of PAS in PAC/PACG eyes is presumed to occur due to the prolonged contact of the peripheral iris with the trabecular meshwork, resulting in closure of the angles and elevation of IOP. In this cross-sectional study, the presence and extent of PAS in PACG eyes correlated with a higher presenting IOP and disease severity. More PAS can lead to a greater reduction in aqueous outflow, and hence higher IOP and in turn more significant visual field losses. The presence of a significant amount of PAS and a higher presenting IOP have been suggested to be predictors of the severity of visual field damage, inadequate IOP control post-LPI, and the need of subsequent surgical interventions^23–27^.

An alternative hypothesis for the development of PAS could be an underlying subclinical inflammation. Whereas PAS formation is typically associated with uveitis and acute primary angle closure (APAC), it is also observed in asymptomatic PAC and chronic PACG eyes (albeit of a narrower width and extent compared to APAC). This signifies the possibility of the presence of low-grade inflammation in PAC/PACG eyes even though aqueous cells and flare are not normally observed clinically at the slit-lamp. Using laser flare cell photometry (Kowa Co, Ltd, Tokyo, Japan), Kong et al.^27,28^ quantitatively assessed the intraocular inflammation in eyes with PACG (with no history of previous APAC) and APAC, and compared them with normal eyes. They found that the mean flare value and mean cell counts were highest in APAC eyes, followed by PACG and normal eyes respectively. They also noted that the mean cell count and flare in both the APAC and PACG groups were significantly higher than those in the normal group^27,28^. Some studies have also demonstrated a higher level of inflammatory cytokines in the aqueous humour of PACG patients compared to normal^27–30^. Ultrastructural evaluation of the trabecular meshwork of chronic PACG eyes revealed the presence of macrophages, leucocytes and inflammatory cells^30–32^. Further investigations are warranted to better elucidate the possible inflammatory component in the development of PAS as it could provide avenues to investigate novel therapeutic targets for PACG such as immunomodulatory therapy. Even if such therapies do not reduce IOP, they may have a complementary role to conventional IOP lowering drugs.

The strength of this study was its relatively large sample size. However, there were several limitations. The patients in our study were predominantly Chinese, and hence our results may not be extrapolated to other populations and separate evaluation in other ethnic groups will be required. Only static anterior segment parameters were evaluated by ASOCT in this study and only one horizontal cross section (nasal to temporal) image was analysed. Therefore, we could not specifically assess whether the predominant localization of PAS had any influence on anterior segment parameters. Dynamic changes, for example changes in iris parameters in response to changes in background illumination may be associated with the development and extent of PAS and should be considered in further studies^32–34^. Qualitative UBM features such as prominent iris roll, iris insertion and basal iris thickness should also be evaluated. The cross-sectional nature of the study makes it difficult to determine any temporal or causal relationship between parameters. Gonioscopy was also only performed once at the time of recruitment to evaluate the presence and extent of PAS. Several studies have shown that the magnitude of PAS can increase, even after LPI^34–36^. The location of LPI may have also impacted PAS development and/or expansion. However, data on the extent of PAS pre-LPI as well as site of LPI were not available. Finally, we excluded subjects with previous surgery such as trabeculectomy or lens extraction with/without goniosynechiolysis in order to exclude changes in anterior segment anatomy caused by surgery. However, this may have led to the inadvertent inclusion of mainly cases of PACG that were responsive to medical therapy.

In summary, our study revealed that almost half of the subjects with PACG demonstrated PAS and with a greater proportion in patients with severe disease. The extent of PAS correlated significantly with presenting IOP and narrower anterior chamber angles. Future research involving longitudinal study designs are required to investigate PAS progression, and how this may influence IOP stability, glaucoma progression and the need for further surgical interventions.

## Supplementary Information

Supplementary Information.

## Data Availability

The authors confirm that the data supporting the findings of this study are available within the article and its supplementary materials.
