# Supplementary material for: Association of peripheral anterior synechiae with anterior segment parameters in eyes with primary angle closure glaucoma
Source: Sci Rep. 2021 Jul 6;11:13906. doi: 10.1038/s41598-021-93293-7 (PMC8260708; doi:10.1038/s41598-021-93293-7)
Supplement: Supplementary file 1 — Supplementary Information. [file 41598_2021_93293_MOESM1_ESM.docx]

| **Table S1: Correlation between the extent of peripheral anterior synechiae and other variables in eyes with primary angle closure glaucoma** | | | | | | | |
| --- | --- | --- | --- | --- | --- | --- | --- |
|  | Presenting IOP | AOD750 | ACD | ACW | ACA | LV | Visual field MD |
| Pearson correlation | 0.43 | -0.29 | -0.16 | -0.03 | -0.15 | 0.18 | -0.23 |
| p-value | <0.001 | <0.001 | 0.02 | 0.63 | 0.03 | 0.007 | <0.001 |
| IOP: intraocular pressure; AOD750: angle opening distance at 750 µm from the scleral spur; ACD: anterior chamber depth; ACW: anterior chamber width; ACA: anterior chamber area; LV: lens vault; MD: visual field mean deviation | | | | | | | |
